# Strength Exercise Confers Protection in Central Nervous System Autoimmunity by Altering the Gut Microbiota

**DOI:** 10.3389/fimmu.2021.628629

**Published:** 2021-03-16

**Authors:** Hao Chen, Liping Shen, Yingying Liu, Xiaomeng Ma, Ling Long, Xueying Ma, Lili Ma, Zhaoyu Chen, Xiuli Lin, Lei Si, Xiaohong Chen

**Affiliations:** Department of Neurology and Multiple Sclerosis Research Center, The Third Affiliated Hospital, Sun Yat-sen University, Guangzhou, China

**Keywords:** exercise, gut microbiota, FMT, EAE, Th17/Treg 3

## Abstract

Exercise therapy including endurance training and resistance training is a promising non-pharmacological therapy in patients with multiple sclerosis (MS). Recent studies have revealed that exercise exerts beneficial impacts on gut microbiota. However, the role of gut microbiota in the immune benefits of strength exercise (SE; one of resistance training) in central nervous system (CNS) autoimmunity is barely known. Here, we observed that 60-min SE ameliorated disease severity and neuropathology in experimental autoimmune encephalomyelitis (EAE), an animal model of MS. SE increased the abundance and diversity of the gut microbiota, and decreased *Firmicutes*/*Bacteroidetes* ratio (F/B ratio) and intestinal mucosal permeability, and enrichment of several short-chain fatty acid (SCFA)-producing bacteria. Furthermore, SE reduced Th17 responses and increased Treg responses in the small intestine lymphoid tissues. Compared to the control group, microbiota-depleted mice receiving SE microbiome fecal transplants had lower disease severity and neuropathology scores. These results uncovered a protective role of SE in neuroimmunomodulation effects partly *via* changes to the gut microbiome.

## Introduction

Multiple sclerosis (MS) is a chronic neurological autoimmune disease that leads to the degeneration of the brain, and the incidence continues to increase worldwide (1). Its pathogenesis remains unclear, and no medication can fully prevent or reverse the clinical progress. In the past decades, genetic predisposition has been linked to many autoimmune diseases, but could not fully explain the pathogenesis of them (2). Environmental factors, such as low vitamin D levels, obesity, smoking, and virus infections, have been related to the increasing incidence of MS (3). Moreover, gut microbiota have been considered as a potential factor contributing to MS epidemiology (4). Experimental autoimmune encephalomyelitis (EAE) mice, an animal model for the human MS, with depleted gut microbiota showed decreased disease severity and neuropathology, which suggests a key role of intestinal flora in EAE mice. Further investigations also proved that gut-residing segmented filamentous bacterium antigens, which presented by dendritic cells, could induce Th17 cell differentiation (5, 6).

Exercise is a cost-effective lifestyle intervention that has the potential to induce various physiological and functional beneficial effects in patients with MS (7). Generally speaking, exercise can be divided into endurance- or strength-based training, which can be enhanced with prolonged exercise training (8). Endurance exercise is aerobic training, including swimming, cycling, or running, whereas strength exercise (SE) is resistance training that includes climbing and muscle training. Various clinical trials demonstrated the safety and effects of exercise training (endurance training and resistance training) (9, 10). Exercise has a positive impact on most of the important functional and health-related factors in MS, such as muscle strength, depression, fatigue, muscle activation, and so on (11). The previous studies examined the neuroprotective effects of exercise training on EAE with conflicting results (12–14). It remains unknown whether exercise mediates effects in EAE by modulating the systemic immune or direct central nervous system (CNS) neuroprotection effects. In addition, exercise can also exert beneficial impacts on gut microbiota. Clarke et al. (15) first discovered that exercise increases gut microbial diversity in humans. Recently, Liu et al. (16) identified the gut microbiota characteristics in patients with prediabetes for improving glucose metabolism and insulin sensitivity through exercise. This provides a key connection between gastrointestinal microbiota and exercise in the development of diabetes. However, what alterations in gut microbiota are involved in the immune benefits of SE in CNS autoimmunity remains obscure.

To address this issue, we undertook this study of SE in the EAE model and showed that SE ameliorated EAE mice at least in part *via* altering gut microbiota. First, we examined the clinical and neuropathological effects of SE in EAE. Then, 16S ribosomal RNA (16S rRNA) gene sequence was used to detect the changes in gut microbiome. Moreover, we found altered T cells in the gut with a reduction of Th17 response and an increase in regulatory T cells. Lastly, we transferred the gut microbiota from mice on SE into microbiota-depleted mice to test whether this protection effects were transmissible *via* their gut microbiome.

## Materials and Methods

### Mice, Instrument, and Reagents

C57BL/6J WT female mice at 3–4 weeks old were purchased from Guangdong Medical Laboratory Animal Center (Guangzhou, China). Before the test, mice were allowed to adapt to the laboratory environment for 1 week. All experiments were performed under guidelines for animal care, according to the National Institutes of Health Guide for Care and Use of Laboratory Animals. All experiments were approved by the Bioethics Committee of South China Agricultural University (Approval ID: 2019-D022). Mice were maintained under specific pathogen-free conditions at South China Agricultural University (Guangzhou, China). Mouse wheel fatigue tester YLS-10B was purchased from Shanghai Yuyan Scientific Instrument Co. Ltd. MOG35-55 peptide (MEVGWYRSPFSRVVHLYRNGK) was synthesized by C.L. Bio-Science Co., Ltd. (Xi'an, China). Pertussis toxin (PTX) was bought from Alexis Corp. (San Diego, CA, USA) and mycobacterium tuberculosis H37RA from Difco Laboratories Inc. (Detroit, MI, USA). Amino acid analysis and mass spectroscopy were conducted to analyze amino acid sequences, and the purity of the peptide was >95%. fluorescein isothiocyanate (FITC)-conjugated anti-mouse CD4, BV421-conjugated anti-mouse interleukin-17A (IL-17A), P-phycoerythrin (PE)-conjugated anti-mouse granulocyte-macrophage colony-stimulating factor (GM-CSF), and PE-conjugated anti-mouse Foxp3 were from BioLegend (San Diego, CA, USA). Metronidazole, vancomycin, neomycin, and ampicillin were purchased from Sigma-Aldrich (San Diego, CA, USA).

### Strength Exercise Training Design

Strength exercise was performed with the wheel fatigue tester designed for mice. After 1-week acclimation, mice (4–5 weeks old) were randomly assigned to four groups: EAE mice (control group), 20 min SE–EAE mice, 40 min SE–EAE mice, and 60 min SE–EAE mice. The running speed for each mouse was according to the instrument guidelines. Mice were familiarized with stair climbing for 10–15 min on 3 consecutive days before the administration of exercise tests and training program. Then, mice in the SE group underwent a different training program (20 min/40 min/60 min 1 and 6 days, 1 week, total 4 weeks), while the *ad libitum* mice group had unrestricted access to run. In order to avoid laziness in mice, an electrified grid that delivered a shock stimulus to stationary mice (0.2–0.4 mA) was installed under each wheel track. When the mice were still stationary after electrical stimulation in the training, they would be given 5 min of rest before running again. Following 4 weeks of the SE training, mice were immunized to induce EAE, and then SE training was continued for 7 days post-immunization.

### Experimental Autoimmune Encephalomyelitis Induction and Evaluation

Experimental autoimmune encephalomyelitis was induced according to the protocol described previously (17). Following 4 weeks on the assigned SE time or *ad libitum*, mice were subcutaneous injected with 300 μg of MOG35-55 peptide emulsified in complete Freund's adjuvant (CFA) that contained 500 μg of mycobacterium tuberculosis H37RA per mice. After 48 h, all mice were intraperitoneal (i.p.) injected 300 ng of PTX in 100 μl of PBS. MOG35-55 peptide in CFA was delivered as an additional immunization 7 days later. After immunization, clinical severity of mice was assessed by a disease severity scale, scoring on a five-point scale: 0, no symptom; 1, tail weakness; 2, paraparesis; 3, paraplegia; 4, paraplegia accompanying with forelimb weakness or paralysis; and 5, moribund animal or death (18).

### Histological and Immunohistochemistry Evaluation

Heart perfusion was conducted in different groups of mice with 4% (w/v) paraformaldehyde after 21 days post-immunization, and their lumbosacral spinal cords were embedded in paraffin. To evaluate the inflammatory infiltration and demyelination, paraffin sections were stained with hematoxylin and eosin (H&E) or with Luxol fast blue (LFB). The inflammation was scored as follows (19): 0, no inflammatory cells; 1, a few scattered inflammatory cells; 2, organization of inflammatory infiltrates around the blood vessels; and 3, extensive perivascular cuffing with extension into the adjacent parenchyma, or parenchymal infiltration without obvious cuffing. Demyelination of the spinal cord was scored as follows (20): 1, traces of subpial demyelination; 2, marked subpial and perivascular demyelination; 3, confluent perivascular or subpial demyelination; 4, massive perivascular and subpial demyelination involving one half of the spinal cord with the presence of cell infiltration into CNS parenchyma; and 5, extensive perivascular and subpial demyelination involving the whole cord section with cell infiltration into the CNS parenchyma. Antibodies to myelin basic protein (MBP) and non-phosphorylated neurofilaments (clone SMI-32) were used to double stain the demyelinated axons and injured axons and analyzed with the positive-staining percentage (number of positive pixels/1 mm^2^).

### Serum ELISA

Serum samples were collected before SE training or *ad libitum* as baseline (T1), after 4 weeks of SE training or *ad libitum* and before EAE immunization (T2) and EAE time (T3). Samples were timely frozen at −80°C until D-lactate (D-Lac), lipopolysaccharide (LPS), and diamine oxidase (DAO) assays were performed. Serum D-Lac, LPS, and DAO concentrations, markers of intestinal permeability damage, were tested using commercially available Enzyme-Linked Immunosorbent Assay Kits (Cloud-Clone Corp., Wuhan, China) according to the manufacturer's instructions.

### Fecal Microbiota Transplantation

About 4- to 5-week-old C57BL/6 female mice were treated with a cocktail of antibiotics (per 1 L of water: metronidazole 1 g, vancomycin 500 mg, and neomycin 1 g, ampicillin 1 g) as microbiota-depleted mice and then used as a recipient of fecal microbiota transplantation (FMT). Antibiotic treatment was administered in the drinking water for 2 weeks. The clearance of intestinal flora was verified by 16S rRNA sequencing analysis. In brief, fresh feces were collected from mice maintained on SE or *ad libitum* for 4 weeks and homogenized in sterile PBS under anaerobic conditions (in carbon dioxide ice) at 5 mg/ml. For FMT, fecal matter from mice of SE training or *ad libitum* for 4 weeks was administered twice per day (200 μl per time) for 2 weeks before EAE induction. Then, recipient mice were immunized with MOG35-55 to induce EAE.

### Gut Microbiota Analysis

Fresh fecal matter was collected and immediately frozen in the carbon dioxide ice for extraction of DNA. Stool DNA extraction was conducted using the QuickGene DNA Tissue Kit from Kurabo Company (Neyagawa, Japan) and PCR amplification. The V3 and V4 regions of microbial 16S rRNA were sequenced by the Illumina MiSeq Technology at BGI Co. (Shenzhen, China). The bioinformatics analysis rarified the OTU to several metrics and evaluated as described previously (21). For alpha diversity analysis, we rarified the OTU to several metrics, including curves of OUT rank, rare faction, calculated indexes of observed species, and Shannon and Simpson indices. For beta diversity analysis, principal component analysis (PCA) was performed using QIIME. The linear discriminant analysis (LDA) effect size (LEfSe) analysis was performed for the quantitative analysis of biomarkers among each group. Briefly, the LEfSe analysis, LDA threshold of > 4, used the non-parametric factorial Kruskal–Wallis (KW) sum-rank test and then used the (unpaired) Wilcoxon rank-sum test to identify the most differently abundant taxa (22).

### Small Intestine LPL Isolation

Small intestine was dissected, and Payer's patches on the epithelial layers were removed by incubating twice with 1 mM dithiothreitol (DTT) and 5 mM ethylenediamine tetraacetic acid (EDTA) in PBS. After 20-min digestion with 1 mg/ml collagenase (type VIII, Sigma-Aldrich) and 300 μg/ml DNase I (Sigma-Aldrich) at 37°C, total lamina propria cells were purified on 40/70% Percoll gradient.

### Flow Staining

To stain the intracellular cytokine, lymphocytes were isolated from the peripheral lymph nodes (LN), spleen, and small intestine lamina propria 21 days after immunization. They were stimulated, fixed, and permeabilized, followed by fluorescent-conjugated intracellular cytokine antibody staining. The Foxp3 Staining Buffer Set (BioLegend) was used to stain the intranuclear Foxp3.

### Statistical Analysis

The experimental autoimmune encephalomyelitis clinical score was expressed as mean ± SEM, whereas other data were expressed as mean ± SD. Two-tailed Student's *t-*test or the non-parametric Mann–Whitney test was performed to analyze the differences between the two groups. Non-parametric data were analyzed using the KW test. Statistical differences were considered significant at *p* < 0.05 level and indicated with asterisks (^*^p < 0.05, ^**^p < 0.01, ^***^p < 0.001, and ^****^p < 0.0001).

## Results

### Strength Exercise Ameliorated the Pathogenesis of EAE

**Figure 1:**
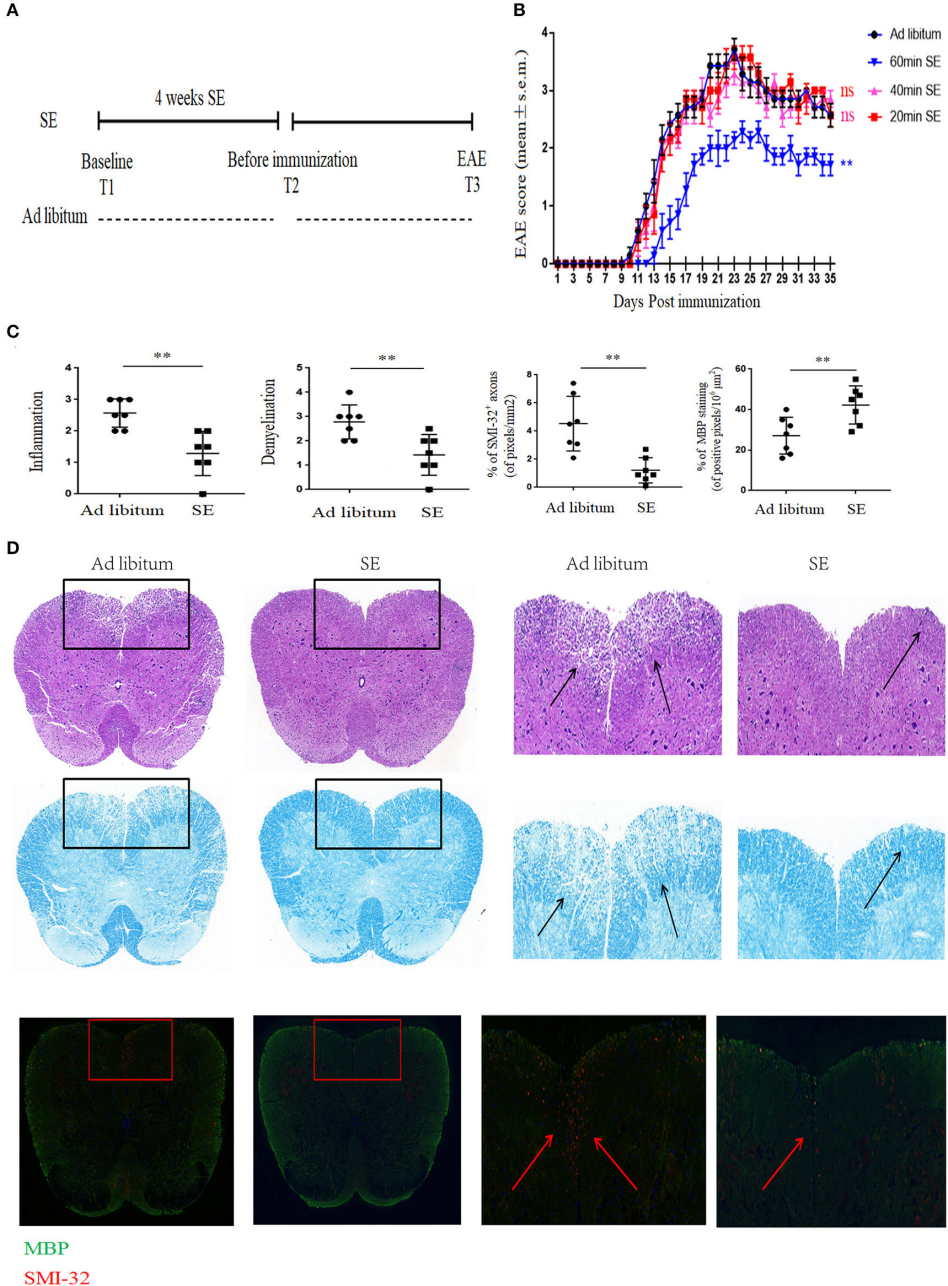
Strength exercise ameliorated clinical severity and neuropathology of experimental autoimmune encephalomyelitis mice. C57BL/6 mice underwent SE or *ad libitum* (control group) for 4 weeks before immunization. **(A)** Timeline for the experimental procedures at baseline before starting SE (T1), after 4 weeks SE, but before immunization (T2), clinical EAE (T3). **(B)** EAE Clinical score of a representative experiment was assessed daily and shown (*n* = 7). Three independent EAE experiments were performed with similar results. **(C)** Histopathology assessment (*n* = 7): at 21 days post-immunization, lumbosacral spinal cords were isolated and performed hematoxylin and eosin (H&E) staining, Luxol fast blue staining or immunostaining for SMI-32^+^ damaged axons (in red) and myelin basic protein (MBP; in green). Scale bars, 200 μm. **(D)** Quantification of inflammation, demyelination, MBP staining, and axonal damage (evaluated by SMI-32^+^ staining) in the lumbosacral spinal cords in the two groups (*n* = 7/group) on day 26 post-immunization. Each dot represents a mouse, and the bars are means ± SD. **p < 0.005.

We used the EAE model to study the effect of SE on CNS autoimmunity. To find the effective time of SE, mice were administrated at different time points of SE training or *ad libitum* (control group) for 4 weeks. The experimental and sample collection timeline is presented in Figure 1A. We found that 20 and 40 min SE training could not exert protective effects on EAE mice. Only 60-min SE training could suppress the severity of EAE. Therefore, 60 min SE training was chosen as the optimal training time for further study. It showed that 60 min SE obviously improved the disease severity, assessed by disease score (Figure 1B). In neuropathology, the SE group had less inflammatory cell infiltration and demyelination (evaluated by H&E and LFB) in the lumber spinal cord (Figures 1C,D). Moreover, SMI-32^+^ damaged axons were reduced, and MBP was increased in the lumber spinal cord of the SE group. And we did not find any obvious adverse effects of SE at the chosen training time.

### Strength Exercise Suppressed Th17 Cells Response and Increased Treg Cells Response in EAE Mice

**Figure 2:**
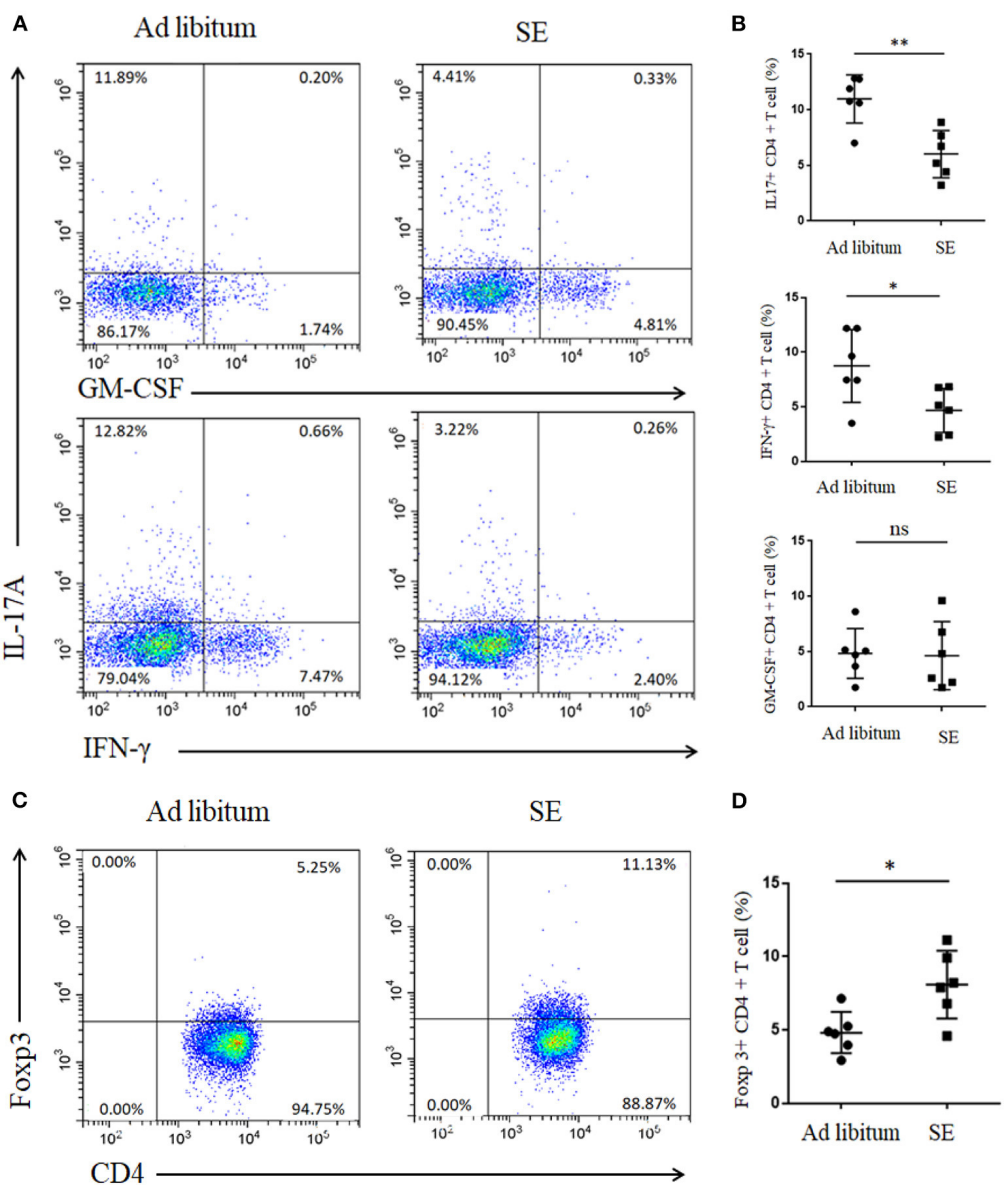
Strength exercise diminish Th17 responses and boost Treg responses in experimental autoimmune encephalomyelitis (T3). Lymphocytes from inguinal lymph nodes (LN) were isolated 21 days post-immunization and used for assessment of different CD4 T-cell subsets (*n* = 6). **(A,C)** Representative staining of different CD4 T-cell subsets in inguinal LN, gated on TCRβ^+^ CD4^+^. **(B,D)** Statistical analysis of the percentages of CD4^+^ T cells producing interleukin-17A (IL-17A), interferon gamma (IFN-γ), granulocyte-macrophage colony-stimulating factor (GM-CSF), and Foxp3. Each dot represents a mouse, and the bars are mean ± SD. *p < 0.05; **p < 0.005; ns, no significance. All *p*-values were calculated by Mann–Whitney test.

Inflammation in the CNS of EAE is caused by aberrant T cells activated in the peripheral lymph nodes draining immunization sites (23). Th17 cells play key roles in EAE with ability to produce proinflammatory cytokines including IL-17A, GM-CSF, and interferon gamma [IFN-γ; (24)]. To investigate whether cell modulation was involved in SE-ameliorating EAE, lymphocytes from inguinal LN were isolated and Th17 cells were detected. CD4^+^ T cells from inguinal LN in SE-training EAE mice produced less IL-17A (*p* < 0.01) and IFN-γ (*p* < 0.05), whereas no significant differences were noted in GM-CSF, which is another pathogenic cytokine of EAE (Figures 2A,B). We also detected the reduced percentage of Treg cells from inguinal LN in SE-training EAE mice (Figures 2C,D). And it has been reported that Treg cells could alleviate EAE by inhibiting functions of Th17 cells (25).

### Strength Exercise Led to Decreased Intestinal Mucosal Permeability

**Figure 3:**
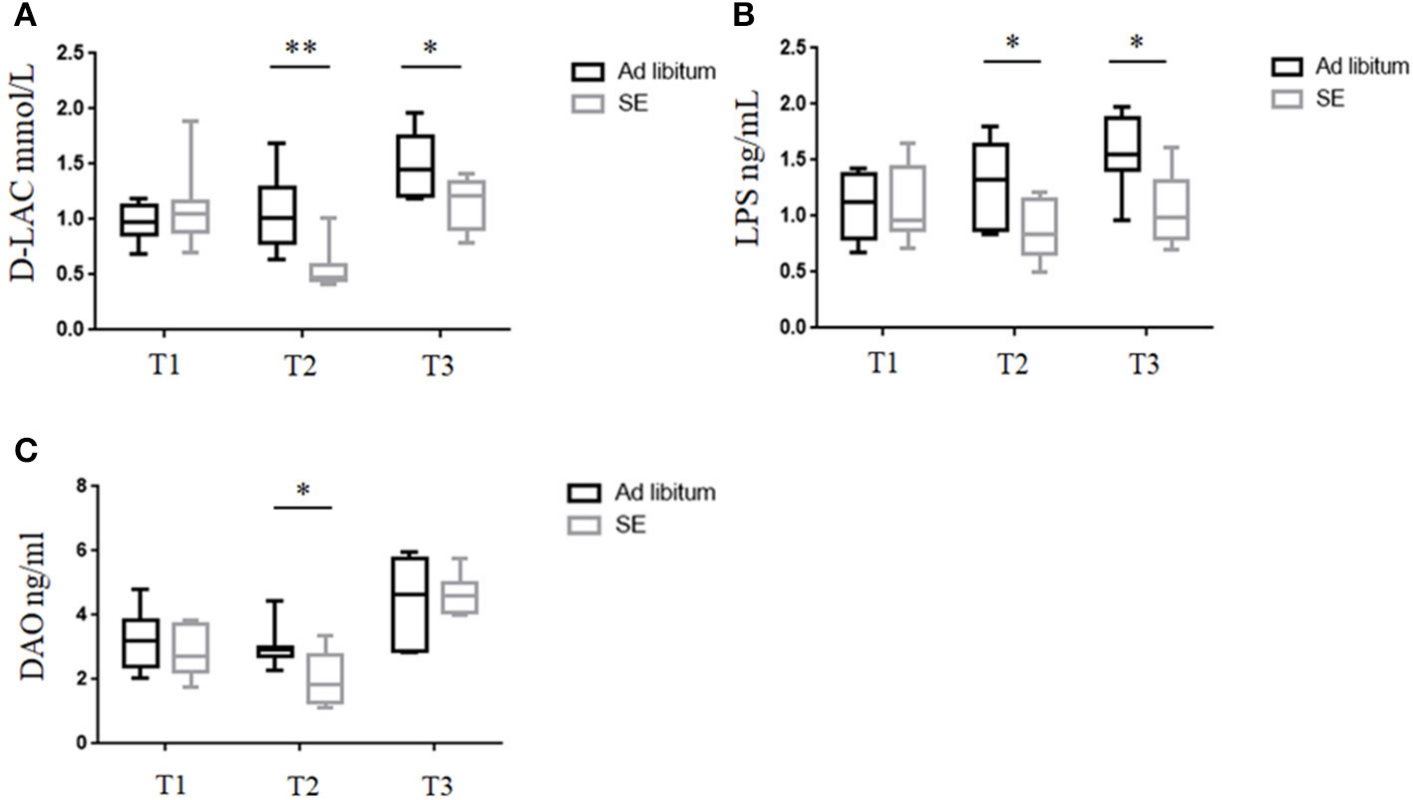
Strength exercise was associated with decreased intestinal permeability damage markers. (*n* = 10) **(A–C)** Serum levels of **(A)** D-lactate (D-lac), **(B)** lipopolysaccharide (LPS), and **(C)** diamine oxidase (DAO) were measured by ELISA at different time points during the experiment. In the ELISA, each sample was run in duplicate. Measurements for D-lac, LPS, and DAO were performed in three independent experiments with similar results. *p < 0.05, **p < 0.01. All *p*-values were calculated by Mann–Whitney test.

The intestinal barrier is important as a selective barrier which could isolate the internal milieu from the microorganism, antigens, and toxins in gut (26). D-lac, DAO, and LPS play important roles in the dysfunction of intestinal barrier (27). Serum was collected from mice at baseline before starting SE (T1), after 4 weeks of SE training but before immunization or *ad libitum* (T2) and EAE time (day 21 post-immunization-T3) (Figure 3). The levels of D-lac, DAO, and LPS in T1, measured by ELISA, were not significantly different in the two groups. Pronounced decreases in serum D-Lac and LPS were observed in SE group relative to the *ad libitum* group levels (Figures 3A,B). Serum DAO levels were significantly decreased in the SE group in T2, but showed no significant difference between the two groups in T3 (Figure 3C).

### Strength Exercise EAE Mice Showed Increased Microbial Diversity and Altered Composition of the Intestinal Microbiome

**Figure 4:**
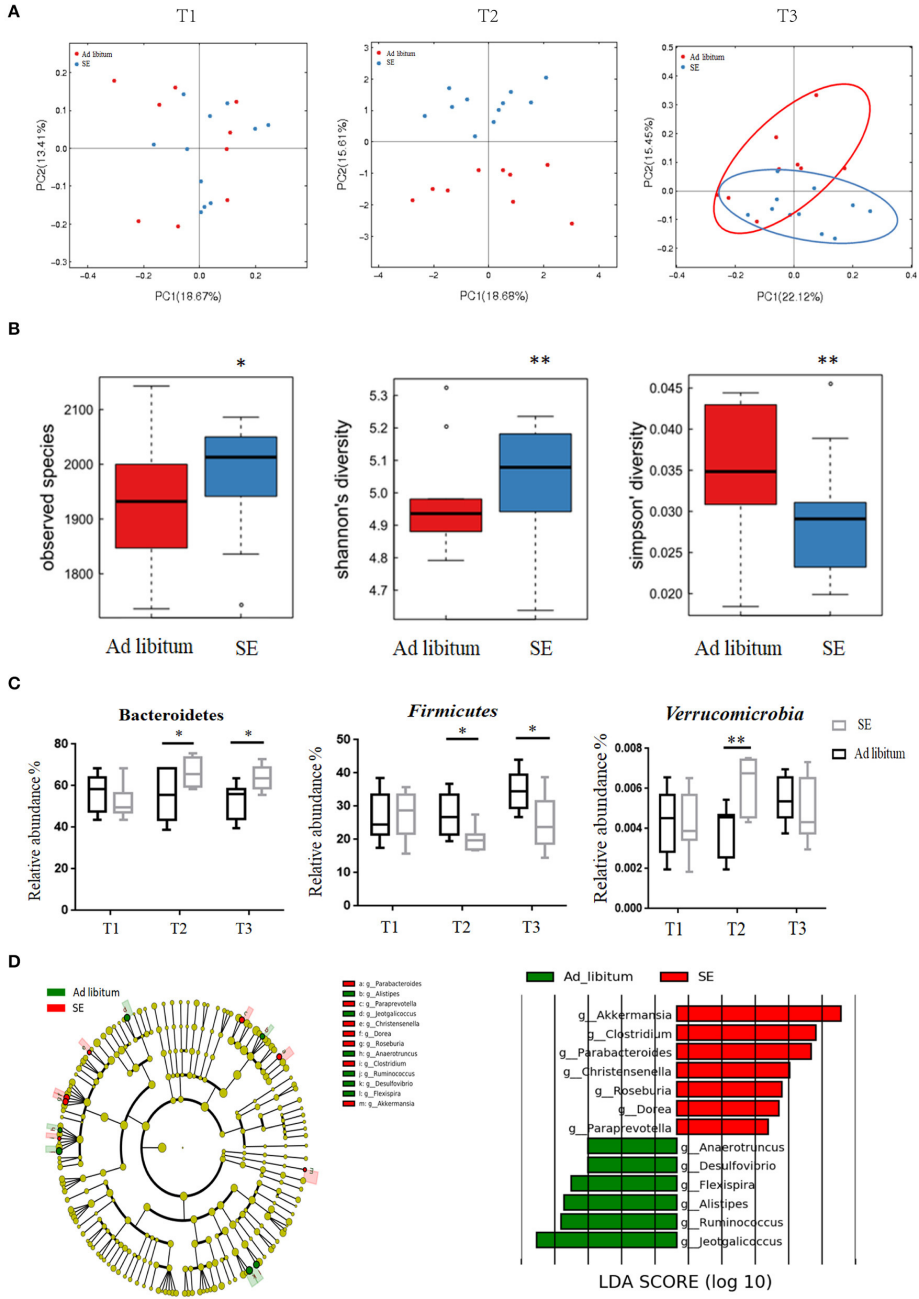
Responses of the diversity, richness, and structure of the gut microbiota on strength exercise in mice. Fecal samples were collected from the SE and *ad libitum* groups at T1 (baseline, *n* = 11 in the SE group, *n* = 9 in the *ad libitum* group), T2 (after 4 weeks on SE training, prior to EAE immunization, *n* = 11 in SE, *n* = 9 in *ad libitum*), and T3 (EAE time, *n* = 11 in SE, *n* = 9 in *ad libitum*). **(A)** Principal coordinate analysis of Bray–Curtis dissimilarity demonstrated the microbiome similarity of SE and *ad libitum* groups at T1, T2, and T3 time points. At T1 time point, fecal samples from two groups indicate similar microbiome (*p* > 0.05, Adonis test). At T2 and T3 time, fecal samples from the two groups indicate two distinct microbiome communities (*p* < 0.05, Adonis test). **(B)** The number of observed OTUs, Shannon's diversity index and Simpson's index values were significantly changed between two groups in T2 time (*p < 0.05; **p < 0.01). **(C)** Bacterial phylum level with significantly different relative abundance between the two groups at T2 and T3. Reported here only those bacterial families with the same direction of difference at T2 and T3 between the two groups (*p < 0.05; **p < 0.01). **(D)** Identification of differential microbes in response to SE in mice based on the linear discriminant analysis (LDA) and effect size (LEfSe) pipeline at T2. Cladogram using LEfSe method indicated the phylogenetic distribution of gut microbiota associated with mice between the two groups. LDA scores showed the significant bacterial differences between the two groups.

Fecal samples were collected at T1, T2, and T3 time points. Gut microbiota analysis between the two groups did not show significant differences at T1 time point, whereas at T2 and T3 time points, the differences were remarkable (Figure 4A). The observed species, as well as the Shannon and Simpson indices were calculated at T2 time point (Figure 4B). Consistent with the number of OTUs, SE increased the richness and diversity of the intestinal microbiota. As presented in Figure 4C, SE training significantly increased the *Bacteroidetes* while decreased the *Firmicutes* at T2 and T3 time points in the phylum level analysis. Furthermore, SE training also significantly increased the relative abundance of *Verrucomicrobia* at T2 time point. In the genus level, the LEfSe analysis score at T2 time point showed bacterial alterations in accordance with the findings the phylum level. In Figure 4D, our results showed that *Akkermansia* within the *Verrucomicrobia* phylum were enriched in the SE group. Decreased abundance of bacteria such as *Anaerotruncus, Jeotgalicoccus, Anaerotruncus, Alistipes, Ruminococcus*, and *Desulfovibrio* and increased abundance in *Clostridium, Parabacteroides, Christensenella, Dorea, Roseburia*, and *Paraprevotella* were observed in the SE group relative to the healthy controls (HCs).

### Strength Exercise Reduced Th17 Cells Response and Increased Tregs Response in the Small Intestine Lymphoid Tissues After 4 Weeks of Training (T2)

**Figure 5:**
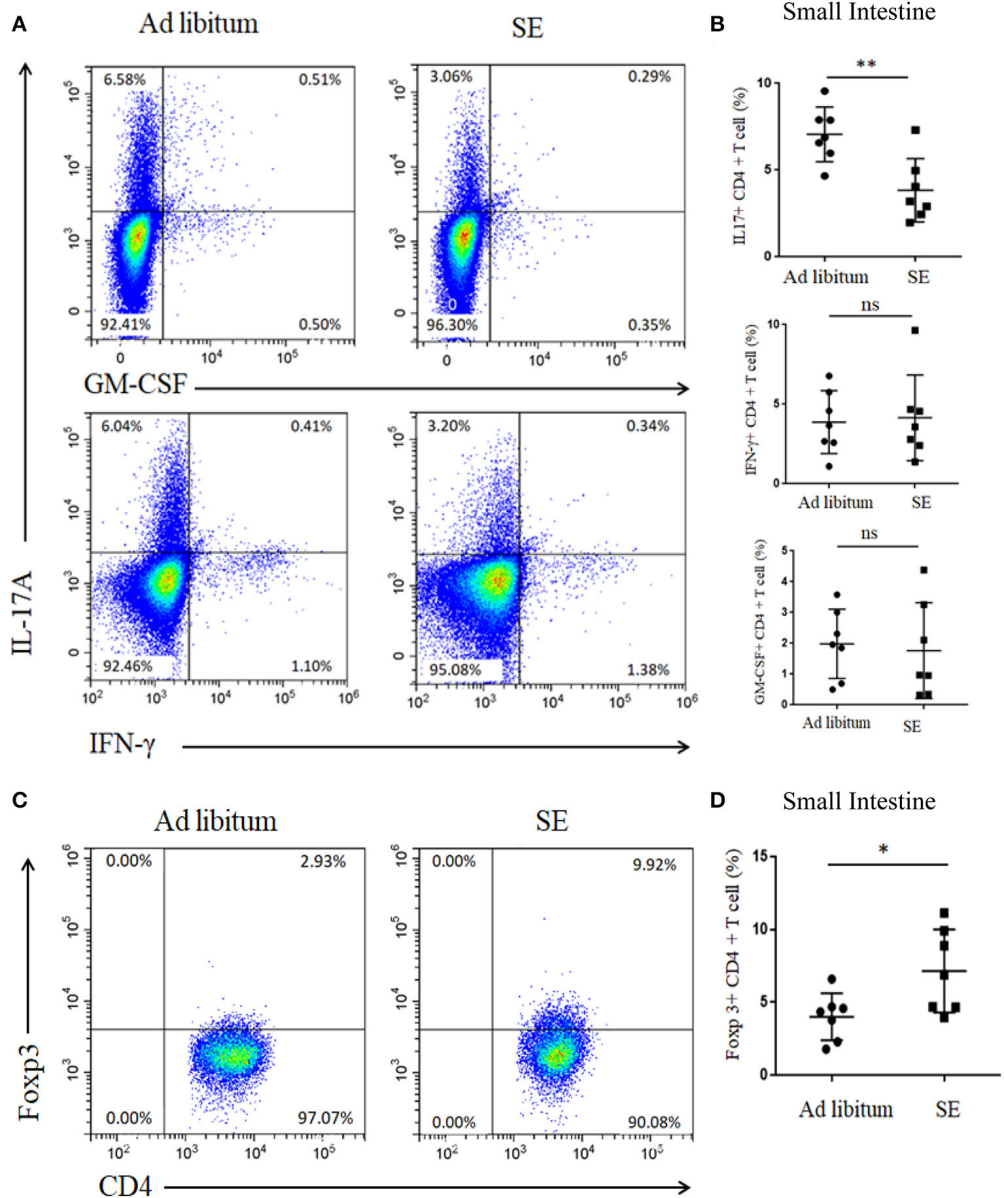
Reduced proportion of IL-17 T cells and increased proportion of Tregs in the small intestine lamina propria after 4 weeks of strength exercise (T2). Lymphocytes from small intestine lamina propria were isolated after 4 weeks of SE without immunization and used for assessment of different CD4 T-cell subsets. **(A,C)** Representative staining of different CD4 T-cell subsets in inguinal lymph nodes (LN), gated on TCRβ^+^ CD4^+^. **(B,D)** Statistical analysis of the percentages of CD4+ T cells producing IL-17A, IFN-γ, GM-CSF, and Foxp3. Each dot represents a mouse, and the bars are mean ± SD. *p < 0.05; **p < 0.005; ns, no significance. All *p*-values were calculated by Mann–Whitney test.

Gut microbiota may directly influence immune cells residing in the gut lymphoid tissue and then modulate local and systemic immune responses (28). Thus, we detected the levels of Th17 and Tregs responses in the small intestine lamina propria after 4 weeks of exercise (T2). In accordance with alterations in microbial community structure of SE, the Th17 response (IL-17) in the small intestine was also decreased in SE mice (Figures 5A,B). However, the percentages of GM-CSF- and IFN-γ-producing T cells were similar in both groups. Moreover, Treg cells increased in the small intestine lamina propria while treatment with SE promoted the Treg differentiation in EAE mice (Figures 5C,D).

### Gut Microbiome Transplantation From SE Mice Confers Neuroprotection in EAE

**Figure 6:**
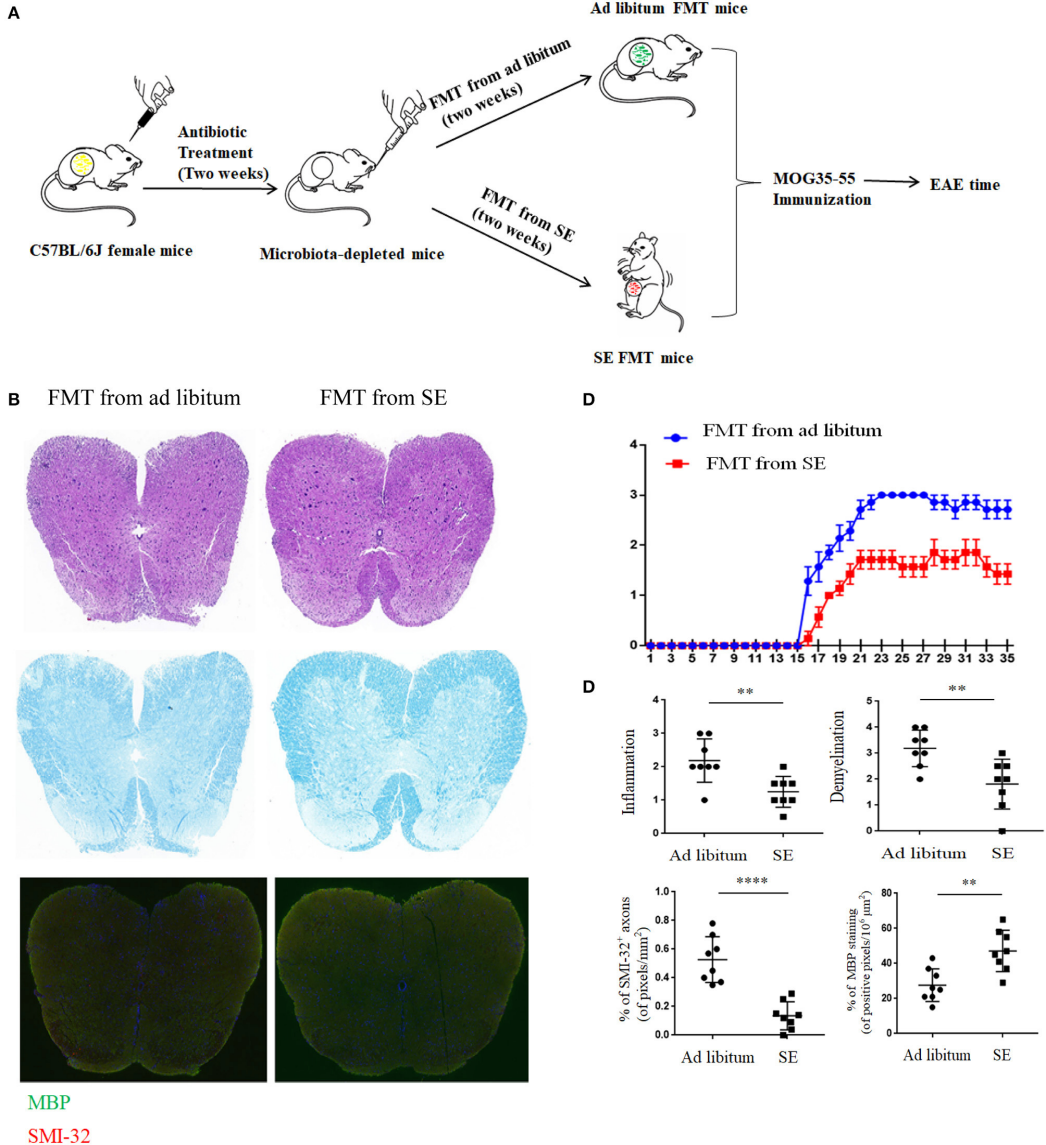
Fecal microbiota transplantation from mice on strength exercise confer protection in experimental autoimmune encephalomyelitis Mice were pretreated with an antibiotic cocktail for 2 weeks and then subjected to FMT from *ad libitum* or SE for 2 weeks before EAE induced. At least two independent EAE experiments were performed with similar results. **(A)** The workflow diagram for FMT study. **(B)** Histopathology assessment (*n* = 8): at 21 days post-immunization, lumbosacral spinal cords were isolated and performed H&E staining, Luxol fast blue staining or immunostaining for SMI-32^+^ damaged axons (in red) and myelin basic protein (MBP; in green). Scale bars, 200 μm. **(C)** EAE clinical score of a representative experiment was assessed daily and shown (*n* = 9). *p* < 0.0001 by two-way ANOVA. **(D)** Quantification of inflammation, demyelination, MBP staining, and axonal damage (evaluated by SMI-32^+^ staining) in the lumbosacral spinal cords in the two FMT groups. Each dot represents a mouse, and the bars are means ± SD. **p < 0.01, ****p < 0.001.

To determine whether the SE-relevant neuroprotection effects are linked with changed gut microbiota, we performed the FMT experiments. In brief, the stool matter from SE mice or *ad libitum* mice was delivered by oral gavage into microbiota-depleted mice that were treated by antibiotic, and then immunized to induce EAE (the workflow diagram for FMT study is presented in Figure 6A). The clearance of intestinal flora was verified by 16S rRNA sequencing analysis (Supplementary Figure S1). The disease severity of recipients with FMT from SE mice was obviously decreased compared with the recipients with FMT from *ad libitum* fed mice (Figure 6C). In spinal cord pathology, SE FMT recipient mice showed decreased lymphocyte infiltration and less demyelination compared to *ad libitum* FMT recipient mice (Figures 6B,D). Moreover, SMI-32^+^ damaged axons were also reduced, and MBP was increased in the lumber spinal cord of SE microbiota recipient mice. Thus, the gut microbiome may play a mechanistic role in the protective effects of SE in EAE.

## Discussion

A congeries of studies have supported the role of exercise in the therapeutic effects of neurological diseases, such as stroke, Parkinson's disease, and Alzheimer's disease (29, 30). Moreover, gut microbiota may play a key role in modulating the oxidative stress and inflammatory responses during endurance exercise (31). However, few reports address the gut microbiota effects of SE on neuroimmune function. Herein, our research first uncovered that SE alleviated the pathogenesis (reduced inflammation, demyelination, and axonal damage) and reduced Th17 responses of EAE, a common animal model of MS. Results of 16S rRNA sequencing and ELISA revealed that SE increased the gut bacterial richness and decreased *Firmicutes*/*Bacteroidetes* ratio (F/B ratio) and intestinal mucosal permeability. Additionally, SE reduced IL-17-producing T cells and increased Tregs cells in the gut lamina propria of normal mice. Notably, FMT from mice on SE into EAE mice had reduced EAE severity and lymphocyte proliferation. Collectively, these results demonstrated that CNS immunomodulatory responses can be mediated by SE-induced changes in the gut microbiota, at least in part.

Accumulating evidence highlights the close interplay between exercise therapy and immune responses (12–14). These animal studies mainly examined the effects of endurance training and systemic immune system on EAE. In human studies, resistance training has been reported to induce effects on muscle strength and functional score after 12 weeks of resistance training (9). Therefore, data from this human study suggest that SE would also be a great exercise pattern for patients with MS. In our report, only 60-min SE training could exert beneficial effects (decrease clinical EAE scores and neuropathology) on EAE mice. The results remind us that SE training on EAE should also take the duration and timing into considerations, which may bring contrast outcomes. We speculate that modifying lifestyle through multiple aspects that correct the imbalance in gut microbiota composition could probably delay the progression of MS, which include adequate sports.

Exercise playing a major role in modulating gut microbiome is well-known (15, 32, 33). Exercise could increase gut microbial diversity and richness in humans. Generally, high diversity is thought to be a marker of healthy status. Low bacterial diversity is recurrently documented in a variety of diseases and is considered to be one of the major types of gut dysbiosis (34). Our current study found that SE also increased bacteria richness and decreased intestinal mucosal permeability. In the phylum level analysis, the *Bacteroidetes* phylum was obviously increased while *Firmicutes* depleted in SE training EAE mice. The two phyla are major microbiome members, which play crucial roles in modulating host inflammation and immune balance (35). Moreover, the elevation of the *F/B* ratio is a sign of proinflammatory status and immune imbalance of autoimmune disorders (36, 37). Meanwhile, the relative high proportion of *Bacteroidetes* to *Firmicutes* can also regulate intestinal epithelium function and reduce inflammation immune responses, which is consistent with our ELISA results. When it comes to the genus level, SE had a striking effect on gut microbiota composition with the enrichment of *Akkermansia, Clostridium, Parabacteroides, Christensenella, Dorea, Roseburia*, and *Paraprevotella*. Most of these genera can produce short-chain fatty acids (SCFAs), including acetate, propionate, and butyrate. SCFAs can stimulate the production of cytokines that are capable of anti-inflammation, such as IL-10 and transforming growth factor-β (TGFβ), which further induce the Treg cell differentiation and regulate the immune responses in the host (38). It may be one of the most important factors by which SE ameliorates EAE. On the other hand, the mechanism behind gut microbiota may be related with MAPK signaling. In our previous study, gut microbiota interventions with *Clostridium butyricum* could reduce CNS-, LN-, small intestine-, colon-, and spleen-infiltration of proinflammatory Th17 cells and increased the percentages of Tregs in the LN, colon, and small intestine. Moreover, the reduced activity of p38 MARK and JNK signaling may contribute to the molecular mechanisms of these benefit effects. Future studies should focus on the molecular mechanisms of the exercise-gut microbiota protection, such as MAPK signaling.

Of note, *Akkermansia*, enriched in the SE group, are commonly used in probiotics for their effects in immunoregulation by converting mucin to SCFAs, anti-inflammation of the adipose tissue by inducing the Treg cells, and suppression of IL-6 and IL-1β (39). However, whether the increased *Akkermansia* could exhibit beneficial effects with mucin degradation is controversial. *Akkermansia* also play a reverse role by inducing intestinal adaptive immune responses during homeostasis (40). The discrepancy suggests that whether *Akkermansia* exert pro- or anti-inflammatory effects may depend on the immune status of the host. The same bacteria can be a double-edged sword in different immune status.

Gut microbiota can modulate the immune responses in a variety of ways, such as decreased differentiation of pathogenic Th1 and Th17 cells and increased differentiation of Treg cells (41). Our study showed that SE led to increased anti-inflammatory Tregs and decreased T cells that produced IL-17 and IFN-γ in the gut and systemically at different times. Th17 cells are regarded as crucial pathogenic cells in EAE, while Treg cells are considered as protective roles by suppressing inflammatory and immune responses. Therefore, the imbalance of T-cell response in this study may lead to the decreased clinical and neuropathological severity in EAE mice. Cignarella et al. (42) also reported that intermittent fasting as a popular lifestyle altered T cells in the gut with a reduction of IL-17-producing T cells and an increase in Treg cells. No significant difference was observed in the GM-CSF analysis, which suggested that the GM-CSF-producing Th17 cell may not be affected by SE. Furthermore, the intestinal mucosal permeability and LPS was decreased by SE, which suggests that the gut microbiota changed by SE also regulate the mucous membrane barrier function. In contrast, LPS is a major component of the outer membrane in Gram-negative bacteria, which induces a strong proinflammatory innate immune response.

Notably, FMT from mice on SE training could transfer protection from EAE, which provided evidence for the involvement of gut microbiome in the beneficial effect of SE. Regulating gut microbiome may offer a novel way to control immune responses. Our prior work showed that gut microbiota interventions with *Clostridium butyricum* and norfloxacin modulate immune responses in EAE mice (22). Moreover, FMT is an effective and novel manipulating treatment for many intestinal disorders, such as *Clostridium difficile* infection, ulcerative colitis, and irritable bowel syndrome (43). Case reports of FMT have also shown favorable outcomes in three patients with MS having gastrointestinal symptoms (44). Therefore, we could speculate that FMT from sportsmen or fitness men to patients with MS might also be a potential treatment for MS. Furthermore, many factors should be carefully considered in FMT treatment, such as *F*/*B* ratio and current immune environmental status of the host.

In conclusion, our results revealed the SE-induced neuroprotection effect in EAE *via* altering microbiota, which led to decreased Th17 cells and increased Treg cells. SE is an important exercise therapy pattern in the future treatment of MS. Only sufficient exercise time could play a protective role in EAE. The results also remind us that the same bacteria may be a double-edged sword in different immune environmental status of the host. Our present study is the first exploration to examine the complex relationships of exercise, intestinal microbiome, and CNS autoimmunity.

## Data Availability Statement

The data presented in the study are deposited in the NCBI repository, accession number PRJNA699980.

## Ethics Statement

All experiments were approved by the Bioethics Committee of South China Agricultural University (Approval ID: 2019-D022).

## Author Contributions

XC, HC, and LSh designed the experiments. YL, LL, XuM, HC, XL, and ZC performed the experiments. HC and XiM drafted the manuscript. LM, LSi, and XC revised the manuscript. All authors participated in reviewing and editing the manuscript.

## Conflict of Interest

The authors declare that the research was conducted in the absence of any commercial or financial relationships that could be construed as a potential conflict of interest.

## References

[1] Reich DS Lucchinetti CF Calabresi PA. Multiple sclerosis. N Engl J Med. (2018) 378:169–80. 10.1056/NEJMra1401483 29320652 PMC6942519

[2] Sawcer S Hellenthal G Pirinen M Spencer CCA Patsopoulos NA Moutsianas L. Genetic risk and a primary role for cell-mediated immune mechanisms in multiple sclerosis. Nature. (2011) 476:214–9. 10.1038/nature10251 21833088 PMC3182531

[3] Ascherio A. Environmental factors in multiple sclerosis. Expert Rev Neurother. (2013) 13:3–9. 10.1586/14737175.2013.865866 24289836

[4] Berer K Gerdes LA Cekanaviciute E Jia XM Xiao L Xia ZK. Gut microbiota from multiple sclerosis patients enables spontaneous autoimmune encephalomyelitis in mice. Proc Natl Acad Sci USA. (2017) 114:10719–24. 10.1073/pnas.1711233114 28893994 PMC5635914

[5] Lécuyer E Rakotobe S Hélène LG Lebreton C Picard M Juste C. Segmented filamentous bacterium uses secondary and tertiary lymphoid tissues to induce gut IgA and specific T helper 17 cell responses. Immunity. (2014) 40:608–20. 10.1016/j.immuni.2014.03.009 24745335

[6] Yang Y Torchinsky MB Gobert M Xiong HZ Xu M Linehan JL. Focused specificity of intestinal TH17 cells towards commensal bacterial antigens. Nature. (2014) 510:152–6. 10.1038/nature13279 24739972 PMC4128479

[7] Motl RW Pilutti LA. The benefits of exercise training in multiple sclerosis. Nat Rev Neurol. (2012) 8:487–97. 10.1038/nrneurol.2012.136 22825702

[8] Hughes DC Ellefsen S Baar K. Adaptations to endurance and strength training. Cold Spring Harb Perspect Med. (2018) 8:a029769. 10.1101/cshperspect.a029769 28490537 PMC5983157

[9] Dalgas U Stenager E Jakobsen J Petersen T Hansen HJ Knudsen C. Resistance training improves muscle strength and functional capacity in multiple sclerosis. Neurology. (2009) 73:1478–84. 10.1212/WNL.0b013e3181bf98b4 19884575

[10] Pearson M Dieberg G Smart N. Exercise as a therapy for improvement of walking ability in adults with multiple sclerosis: a meta-analysis. Arch Phys Med Rehabil. (2015) 96:1339–48.e1337. 10.1016/j.apmr.2015.02.011 25712347

[11] Dalgas U Stenager E Ingemann HT. Multiple sclerosis and physical exercise: recommendations for the application of resistance-, endurance- and combined training. Mult Scler. (2008) 14:35–53. 10.1177/1352458507079445 17881393

[12] Souza PS Gonçalves ED Pedroso GS Farias HR Junqueira SC Marcon R. Physical exercise attenuates experimental autoimmune encephalomyelitis by inhibiting peripheral immune response and blood-brain barrier disruption. Mol Neurobiol. (2017) 54:4723–37. 10.1007/s12035-016-0014-0 27447807

[13] Einstein O Fainstein N Touloumi O Lagoudaki R Hanya E Grigoriadis N. Exercise training attenuates experimental autoimmune encephalomyelitis by peripheral immunomodulation rather than direct neuroprotection. Exp Neurol. (2018) 299:56–64. 10.1016/j.expneurol.2017.10.008 29031957

[14] Pryor WM Freeman KG Larson RD Edwards GL White LJ. Chronic exercise confers neuroprotection in experimental autoimmune encephalomyelitis. J Neurosci Res. (2015) 93:697–706. 10.1002/jnr.23528 25510644

[15] Clarke SF Murphy EF O'Sullivan O Lucey AJ Humphreys M Hogan A. Exercise and associated dietary extremes impact on gut microbial diversity. Gut. (2014) 63:1913–20. 10.1136/gutjnl-2013-306541 25021423

[16] Liu Y Wang Y Ni Y Cheung CKY Lam KSL Wang Y. Gut microbiome fermentation determines the efficacy of exercise for diabetes prevention. Cell Metab. (2020) 31:77–91.e75. 10.1016/j.cmet.2019.11.001 31786155

[17] Ying J Yan Z Shaoqiong C Cansheng Zhu Aimin Wu Yingying Liu. The anti-inflammatory effect of donepezil on experimental autoimmune encephalomyelitis in C57 BL/6 mice. Neuropharmacology. (2013) 73:415–24. 10.1016/j.neuropharm.2013.06.023 23831366

[18] Mycko MP Sliwinska B Cichalewska M Cwiklinska H Raine CS Selmaj KW. Brain glycolipids suppress T helper cells and inhibit autoimmune demyelination. J Neurosci. (2014) 34:8646–58. 10.1523/jneurosci.0885-14.2014 24948818 PMC6608209

[19] O'Neill EJ Day MJ Wraith DC. IL-10 is essential for disease protection following intranasal peptide administration in the C57BL/6 model of EAE. J Neuroimmunol. (2006) 178:1–8. 10.1016/j.jneuroim.2006.05.030 16872684 PMC3399771

[20] Kuerten S Kostova-Bales DA Frenzel LP Tigno JT Tary-Lehmann M Angelov DN. MP4- and MOG:35-55-induced EAE in C57BL/6 mice differentially targets brain, spinal cord and cerebellum. J Neuroimmunol. (2007) 189:31–40. 10.1016/j.jneuroim.2007.06.016 17655940 PMC2083209

[21] Caporaso JG Kuczynski J Stombaugh J Bittinger K Bushman FD Costello EK. QIIME allows analysis of high-throughput community sequencing data. Nat Methods. (2010) 7:335–6. 10.1038/nmeth.f.303 20383131 PMC3156573

[22] Chen H Ma X Liu Y Ma L Chen Z Lin X. Gut microbiota interventions with clostridium butyricum and norfloxacin modulate immune response in experimental autoimmune encephalomyelitis mice. Front Immunol. (2019) 10:1662. 10.3389/fimmu.2019.01662 31428083 PMC6689973

[23] Goverman J. Autoimmune T cell responses in the central nervous system. Nat Rev Immunol. (2009) 9:393–407. 10.1038/nri2550 19444307 PMC2813731

[24] Singh RP Hasan S Sharma S Nagra S Yamaguchi DT Wong DTW. Th17 cells in inflammation and autoimmunity. Autoimmun Rev. (2014) 13:1174–81. 10.1016/j.autrev.2014.08.019 25151974

[25] Kleinewietfeld M Hafler DA. Regulatory T cells in autoimmune neuroinflammation. Immunol Rev. (2014) 259:231–44. 10.1111/imr.12169 24712469 PMC3990868

[26] Wijtten PJ Meulen VDJ Verstegen MW. Intestinal barrier function and absorption in pigs after weaning: a review. Br J Nutr. (2011) 105:967–81. 10.1017/s0007114510005660 21303573

[27] Zhuang S Zhong J Bian YF Fan YS Chen QY Liu P. Rhein ameliorates lipopolysaccharide-induced intestinal barrier injury via modulation of Nrf2 and MAPKs. Life Sci. (2019) 216:168–75. 10.1016/j.lfs.2018.11.048 30471284

[28] Gaboriau-Routhiau V Rakotobe S Lécuyer E Mulder I Lan A Bridonneau C. The key role of segmented filamentous bacteria in the coordinated maturation of gut helper T cell responses. Immunity. (2009) 31:677–89. 10.1016/j.immuni.2009.08.020 19833089

[29] Memon AA Coleman JJ Amara AW. Effects of exercise on sleep in neurodegenerative disease. Neurobiol Dis. (2020) 140:104859. 10.1016/j.nbd.2020.104859 32243913 PMC7497904

[30] Han P Zhang W Kang L Ma YX Fu LY Jia LY. Clinical evidence of exercise benefits for stroke. Adv Exp Med Biol. (2017) 1000:131–51. 10.1007/978-981-10-4304-8_9 29098620

[31] Mach N Fuster-Botella D. Endurance exercise and gut microbiota: a review. J Sport Health Sci. (2017) 6:179–97. 10.1016/j.jshs.2016.05.001 30356594 PMC6188999

[32] Allen JM Mailing LJ Cohrs J Salmonson C Fryer JD Nehra V. Exercise training-induced modification of the gut microbiota persists after microbiota colonization and attenuates the response to chemically-induced colitis in gnotobiotic mice. Gut Microbes. (2018) 9:115–30. 10.1080/19490976.2017.1372077 28862530 PMC5989796

[33] O'Sullivan O Cronin O Clarke SF Murphy EF Molloy MG Shanahan F. Exercise and the microbiota. Gut Microbes. (2015) 6:131–6. 10.1080/19490976.2015.1011875 25800089 PMC4615660

[34] Levy M Kolodziejczyk AA Thaiss CA Elinav E. Dysbiosis and the immune system. Nat Rev Immunol. (2017) 17:219–32. 10.1038/nri.2017.7 28260787

[35] Chang CJ Lin CS Lu CC Martel J Ko YF Ojcius DM. Ganoderma lucidum reduces obesity in mice by modulating the composition of the gut microbiota. Nat Commun. (2015) 6:7489. 10.1038/ncomms8489 26102296 PMC4557287

[36] Ley RE Turnbaugh PJ Klein S Gordon JI. Microbial ecology: human gut microbes associated with obesity. Nature. (2006) 444:1022–3. 10.1038/4441022a 17183309

[37] Ley RE Bäckhed F Turnbaugh P Lozupone CA Knight RD Gordon JI. Obesity alters gut microbial ecology. Proc Natl Acad Sci U S A. (2005) 102:11070–5. 10.1073/pnas.0504978102 16033867 PMC1176910

[38] Smith PM Howitt MR Panikov N Michaud M Gallini CA Bohlooly-Y M. The microbial metabolites, short-chain fatty acids, regulate colonic Treg cell homeostasis. Science. (2013) 341:569–73. 10.1126/science.1241165 23828891 PMC3807819

[39] Anhê FF Roy D Pilon G Dudonné S Matamoros S Varin TV. A polyphenol-rich cranberry extract protects from diet-induced obesity, insulin resistance and intestinal inflammation in association with increased *Akkermansia* spp. population in the gut microbiota of mice. Gut. (2015) 64:872–83. 10.1136/gutjnl-2014-307142 25080446

[40] Ansaldo E Slayden LC Ching KL Koch MA Wolf NK Plichta DR. Akkermansia muciniphila induces intestinal adaptive immune responses during homeostasis. Science. (2019) 364:1179–84. 10.1126/science.aaw7479 31221858 PMC6645389

[41] Chu F Shi M Lang Y Shen D Jin T Zhu J. Gut microbiota in multiple sclerosis and experimental autoimmune encephalomyelitis: current applications and future perspectives. Mediators Inflamm. (2018) 2:8168717. 10.1155/2018/8168717 29805314 PMC5902007

[42] Cignarella F Cantoni C Ghezzi L Salter A Dorsett Y Chen L. Intermittent fasting confers protection in CNS autoimmunity by altering the gut microbiota. Cell Metab. (2018) 27:1222–35.e6. 10.1016/j.cmet.2018.05.006 29874567 PMC6460288

[43] Borody TJ Khoruts A. Fecal microbiota transplantation and emerging applications. Nat Rev Gastroenterol Hepatol. (2011) 9:88–96. 10.1038/nrgastro.2011.244 22183182

[44] Borody TJ Brandt LJ Paramsothy S. Therapeutic faecal microbiota transplantation: current status and future developments. Curr Opin Gastroenterol. (2014) 30:97–105. 10.1097/mog.0000000000000027 24257037 PMC3868025

