# Supplementary figures and images for: Strength Exercise Confers Protection in Central Nervous System Autoimmunity by Altering the Gut Microbiota
Source: Front Immunol. 2021 Mar 16;12:628629. doi: 10.3389/fimmu.2021.628629 (PMC8007788; doi:10.3389/fimmu.2021.628629)

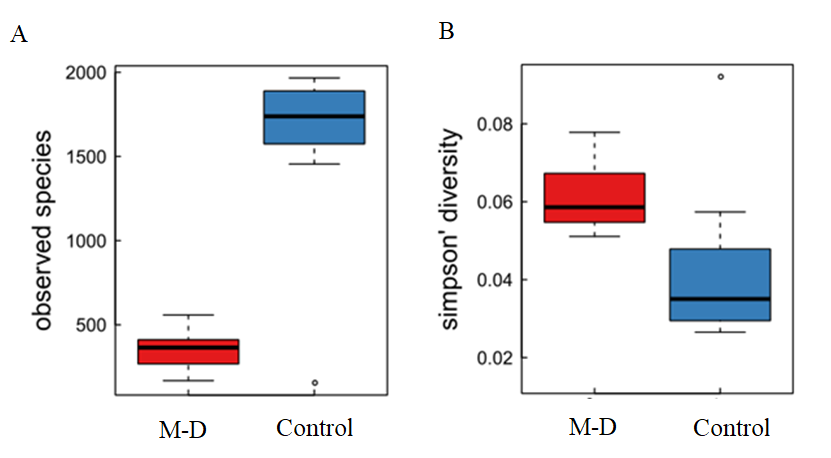

Supplement: Supplementary file 1 [file Image_1.TIF]
