# Supplementary material for: Strength Exercise Confers Protection in Central Nervous System Autoimmunity by Altering the Gut Microbiota
Source: Front Immunol. 2021 Mar 16;12:628629. doi: 10.3389/fimmu.2021.628629 (PMC8007788; doi:10.3389/fimmu.2021.628629)
Supplement: Supplementary file 2 [file Table_1.DOCX]

**Supplementary Materials**

Figure S1. The clearance of intestinal flora by cocktail antibiotics was verified by 16s rRNA sequencing analysis. (n=8) The number of observed OTUs, shannon diversity index and simpson index values were significantly changed between microbiota-depleted (M-D) group and Control group.
